# Candidate genes in quantitative trait loci associated with absolute and relative kidney weight in rats with Inherited Stress Induced Arterial Hypertension

**DOI:** 10.1186/1471-2156-16-S1-S1

**Published:** 2015-02-02

**Authors:** Olga E Redina, Svetlana E Smolenskaya, Leonid O Klimov, Arcady L Markel

**Affiliations:** 1 Institute of Cytology and Genetics, Siberian Branch of Russian Academy of Sciences, Novosibirsk, 630090 Russia; 2 Novosibirsk State University, Novosibirsk, 630090 Russia

**Keywords:** QTL/microarray approach, absolute and relative kidney weight, arterial blood pressure, candidate genes, stress-induced arterial hypertension, ISIAH rats

## Abstract

**Background:**

The kidney mass is significantly increased in hypertensive ISIAH rats with Inherited Stress Induced Arterial Hypertension as compared with normotensive WAG rats. The QTL/microarray approach was carried out to determine the positional candidate genes in the QTL for absolute and relative kidney weight.

**Results:**

Several known and predicted genes differentially expressed in ISIAH and WAG kidney were mapped to genetic loci associated with the absolute and relative kidney weight in 6-month old F2 hybrid (ISIAHxWAG) males. The knowledge-driven filtering of the list of candidates helped to suggest several positional candidate genes, which may be related to the structural and mass changes in hypertensive ISIAH kidney.

In the current study, we showed that all loci found for absolute and relative kidney weight didn't overlap with significant or suggestive loci for arterial blood pressure level. So, the genes differentially expressed in ISIAH and WAG kidneys and located in these QTL regions associated with absolute and relative kidney weight shouldn't substantially influence the BP level in the 6 month-old ISIAH rats. However, in some cases, small effects may be suggested.

**Conclusions:**

The further experimental validation of causative genes and detection of polymorphisms will provide opportunities to advance our understanding of the underlying nature of structural and mass changes in hypertensive ISIAH kidney.

## Background

Renal function plays a major role in long-term control of arterial blood pressure and sodium balance [1]. Kidney as a target organ in hypertension is widely investigated. Differences in the kidney size have been observed between most rat models of hypertension and their respective normotensive controls [2]. The alterations in kidney size may occur as a consequence of pathophysiological processes underlying the hypertension development. Several studies were conducted in order to find the genetic determinants for hypertensive dependent relative kidney weight changes and several genetic loci associated with this trait were found [2,3]. However little is known about particular genes participating in the trait manifestation.

The use of experimental animal models provides valuable information to elucidate the nature of polygenic traits [4]. The ISIAH (Inherited Stress-Induced Arterial Hypertension) rat strain was developed to study the mechanisms of the stress-induced hypertension and its complications [5]. The ISIAH rats show a number of characteristic features of hypertensive state: the elevated systolic arterial blood pressure (SABP) at basal condition, a dramatic increase in SABP when restrained, hypertrophy of the heart left ventricle, increase in the wall thickness of the small arteries, and changes in the ECG pattern [6]. In addition, ISIAH rats have significantly increased kidney mass as compared to normotensive controls [7].

Earlier we used quantitative trait loci (QTL) approach, which helps to map the genomic regions associated with the phenotypic variation of quantitative physiological traits, and we described several QTL for absolute and relative kidney weight in 6 month old F_2_(ISIAH × WAG) hybrid male rats [8]. Our results suggested that absolute and relative kidney weights are complex phenotypes resulting from a large number of factors, each exhibiting a small effect QTL for hypertension.

The combined use of QTL mapping and subsequent microarray profiling of nonrecombinant parental strains is recognized as a powerful tool to identify the genes underlying QTL [9] and to reduce the number of candidate genes in the QTL regions [10,11].

Earlier we described the results of the comparative analysis of gene expression profiling which revealed differentially expressed genes in kidney of hypertensive ISIAH and normotensive WAG rats. The functional annotation of the genes differentially expressed in ISIAH and WAG kidney helped to suggest the genetic determinants related to blood pressure control in ISIAH rats. The analysis showed that many genes are working in stress-related mode in hypertensive kidney and the alterations in gene expression are likely related to both pathophysiological and compensatory mechanisms [12].

The present work was carried out to determine the differentially expressed genes present in QTL for absolute and relative kidney weight in 6 month old F_2_(ISIAH × WAG) hybrid male rats and related to the mechanisms defining the differences in hypertensive and normotensive kidney weight.

In the current study, several known and predicted genes differentially expressed in ISIAH and WAG kidney were mapped to genetic loci associated with the absolute and relative kidney weight in 6-month old F_2 _hybrid (ISIAHxWAG) males. The knowledge-driven filtering of the list of candidates helped to suggest several positional candidate genes, which may be related to the structural and mass changes in hypertensive ISIAH kidney. Besides, we showed that loci for absolute and relative kidney weight didn't overlap with significant or suggestive loci for arterial blood pressure level. The role of loci with small effects is discussed.

## Methods

### Animals

The hypertensive ISIAH (Inherited Stress Induced Arterial Hypertension) and normotensive WAG (Wistar Albino Glaxo) rats bred in the Laboratory of Experimental Animals at the Institute of Cytology and Genetics (Novosibirsk, Russia) were used. All rats were maintained in the standard conditions with free access to food and water. All animal experiments were approved by the Institute's Animal Care and Use Committee.

The description of animals used in QTL analysis was given earlier [7]. QTL analysis for absolute and relative kidney weight was performed using 6-month old F_2 _hybrid males (n = 126) derived from a cross of ISIAH and WAG rats. The genome scan was carried out with 149 polymorphic microsatellite markers (141 markers were for autosomes and 8 markers were for chromosome X). The list of markers and the genomic coverage data are available on the site of Institute of Cytology and Genetics SB RAS http://icg.nsc.ru/isiah/en/category/qtl/. The relative kidney weight was expressed as the ratio of organ weight to the body weight (g/100 g b.w.).

The 6-month old ISIAH (n = 3), and WAG (n = 3) males were used in microarray experiments. Their SABP was 173.67 ± 1.86 mmHg in ISIAH and 124.67 ± 2.67 mmHg in WAG males. The SABP was measured indirectly by the tail-cuff method. The blood pressure level was determined under short-term ether anesthesia to exclude the effect of psychological stress induced by the measuring procedure. Renal cortex and renal medulla were analyzed separately. The kidney of the decapitated rats was immediately removed and sectioned to get the samples of renal cortex and renal medulla. Samples (50 mg) were homogenized in 1 ml of TRIzol (Invitrogen Life Technologies, USA) in glass homogenizers, removed to 1.5-ml Eppendorf tubes and stored at −70°C until RNA isolation.

**The details of QTL analysis **were described earlier [8,13]. Genomic DNA was prepared from liver by the conventional method using Proteinase K and phenol-chloroform extraction. Isolated genomic DNA was precipitated and dissolved in deionized water. The http://www.ensembl.org/Rattus_norvegicus database was used to define the relative positions of the markers along chromosomes given in Megabases (Mb). Genotyping: 50-100 ng of genomic DNA was amplified by PCR in reaction buffer containing 2 μmol of each primer, 200 μmol of each dNTP, 1.5 mmol MgCl_2 _and 0.2 U of Taq DNA Polymerase (Medigen, Russia). The PCR reactions were performed following the protocol: initial denaturation at 95°C for 3 minutes, followed by 38 cycles of denaturation at 94°C for 20 seconds, annealing for 15 seconds at a temperature specific to each pair of primers and elongation at 72°C for 20 seconds. Cycles were followed by a final extension step at 72°C for 5 minutes. The time of elongation was not varied because all the amplified fragments were shorter than 300 nucleotides. The product of each tube was analyzed by electrophoresis in 6-8% polyacrylamide gel in TBE buffer at 10 V/cm. The separated fragments were visualized by staining with ethidium bromide and analyzed on gel-imager Biometra (Germany).

### Linkage and statistical analysis

The data for relative kidney weight were transformed using natural logarithm to reduce skewness and kurtosis in the distribution. Linkage analysis was done using the MAPMAKER/EXP 3.0 and MAPMAKER/QTL 1.1 programs kindly provided by Dr. Eric Lander (Whitehead Institute, Cambridge, MA) [14]. The chromosome X was analyzed as backcross group. The QTL boundaries were determined in the respective one LOD confidence interval. Position of markers was given in megabases (Mb) according to RGSC Genome Assembly v 5.0.

The QTL Cartographer Version 1.17, JZmapqtl http://statgen.ncsu.edu[15,16] was used to assess genome-wide and chromosome-wise empirical significant threshold values for QTLs. Permutation test was done using 1000 permutations of the original data [17]. The LOD scores exceeding 5% experiment wise threshold value were taken as significant evidence of linkage [18]. LOD scores exceeding 5% chromosome-wise threshold value were considered as suggestive linkage.

### Microarray experiments

The collected samples were sent to JSC Genoanalytica (Moscow, Russia), where total RNA was extracted and processed. Three samples from ISIAH kidney and three samples from WAG kidney were run as experimental replicates. Four hundred nanograms of total RNA was used for complementary RNA in vitro transcription, followed by a T7 RNA polymerase-based linear amplification and labeling with the TotalPrep RNA Labeling Kit using Biotinylated-UTP (Ambion, Austin, TX). The signal was developed by staining with Cy3-streptavidin. The hybridization was performed on Illumina RatRef-12 Expression BeadChip microarray platform containing 22,524 probes for a total of 22, 228 rat genes selected primarily from the National Center for Biotechnology Information RefSeq database (Release 16; Illumina, San Diego, CA, USA). Hybridization, washing and staining were carried out according to the Illumina Gene Expression Direct Hybridization Manual. The BeadChip was scanned on a high-resolution Illumina BeadArray reader.

### Microarray data extraction, normalization, and analyses

The primary statistical analysis of the hybridization results was performed by JSC Genoanalytica (Moscow, Russia). The Illumina GenomeStudio software was used to extract fluorescence intensities and normalize the expression data. Data acquisition and analysis were done using gene expression module and rank invariant normalization. After normalization, genes were filtered by their 'detection' p-value, which had to be less then 0.01 (significantly detected), in both samples. Subsequently, the differentially expressed genes were identified using the Illumina Custom error model, which provides an expression difference score (Diff-Score) taking into account background noise and sample variability. Genes were considered significantly changed at a |Differential Score| of more than 20, which was equivalent to a p-value of less than 0.01. Fold changes were calculated as ratio of gene expression value in ISIAH to gene expression value in WAG. The lists of genes differentially expressed in kidney of hypertensive ISIAH and normotensive WAG rats are available on the site of Institute of Cytology and Genetics SB RAS http://icg.nsc.ru/isiah/en/. Heatmaps were constructed from normalized signals using gplots package for R statistical software http://cran.r-project.org/web/packages/gplots/index.html.

## Results and discussion

Many different reasons may cause the increase of the kidney weight. It may be modified by hypertrophy and/or hyperplasia of the kidney tissues. Each of these processes may be under common and partly separate control and may be triggered also by some common and specific stimuli [19].

The significant positive correlation was shown between kidney weight and glomerular number and size [20]. Comparative electron microscopic study of glomerular apparatus in 6-month old ISIAH and Wistar rats showed hypertrophy of renal corpuscles in hypertensive kidney, accompanied by multiple structural changes such as capillary narrowing or dilation, endothelial flattening, podocyte hypertrophy and flattening of their cytopodia, thickening of basal lamina, mesangial volume expansion and increase in the number of intercapillary processes of mesangial cells [21]. Besides, the renal medullary interstitial cells of ISIAH kidneys were characterized by higher numerical density and were enlarged with a higher volume share of their secretory granules [22]. Complex of these signs suggested a disturbance of glomerular capillary blood circulation and a functional podocyte stress, compensating the microcirculatory disturbances. Changes in basal membranes and mesangium are indicative of not only increase in filtration barrier functional load, but also of initial stages of glomerular [21] and renomedullar sclerosis [22].

The QTL analysis revealed 6 suggestive loci for kidney weight on chromosomes 4, 6, 10, 15, 17, and X. One significant locus on Chr.7 and three suggestive loci on Chr.2, 3, and 6 were found for relative kidney weight. The description of all these loci was done earlier [8].

**Figure 1:**
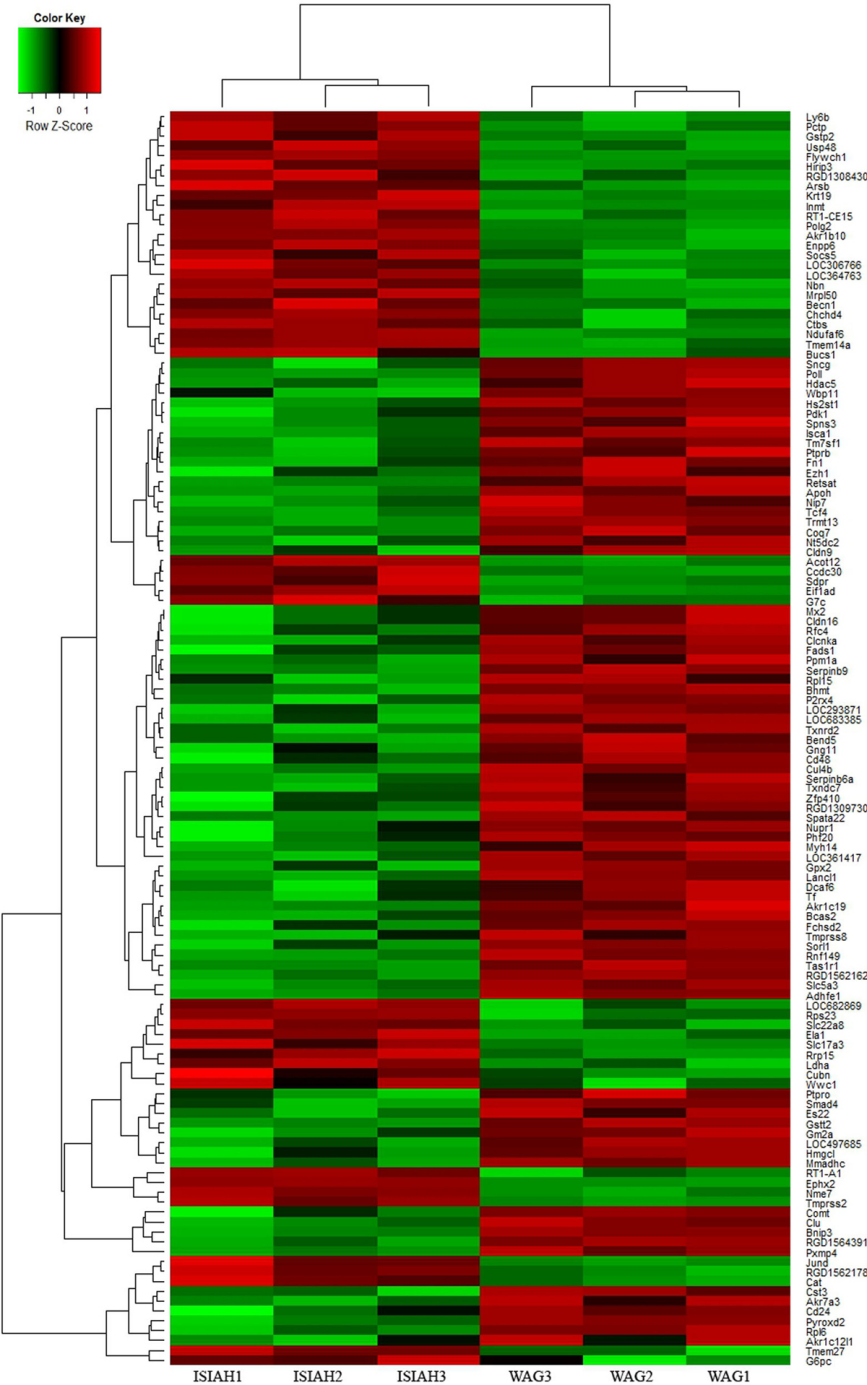
**Hierarchical clustering of the genes differentially expressed in renal cortex of hypertensive ISIAH and normotensive WAG rats**. Normalised gene expression is indicated by the row Z-score where red represents upregulated genes and green represents downregulated genes.

**Figure 2:**
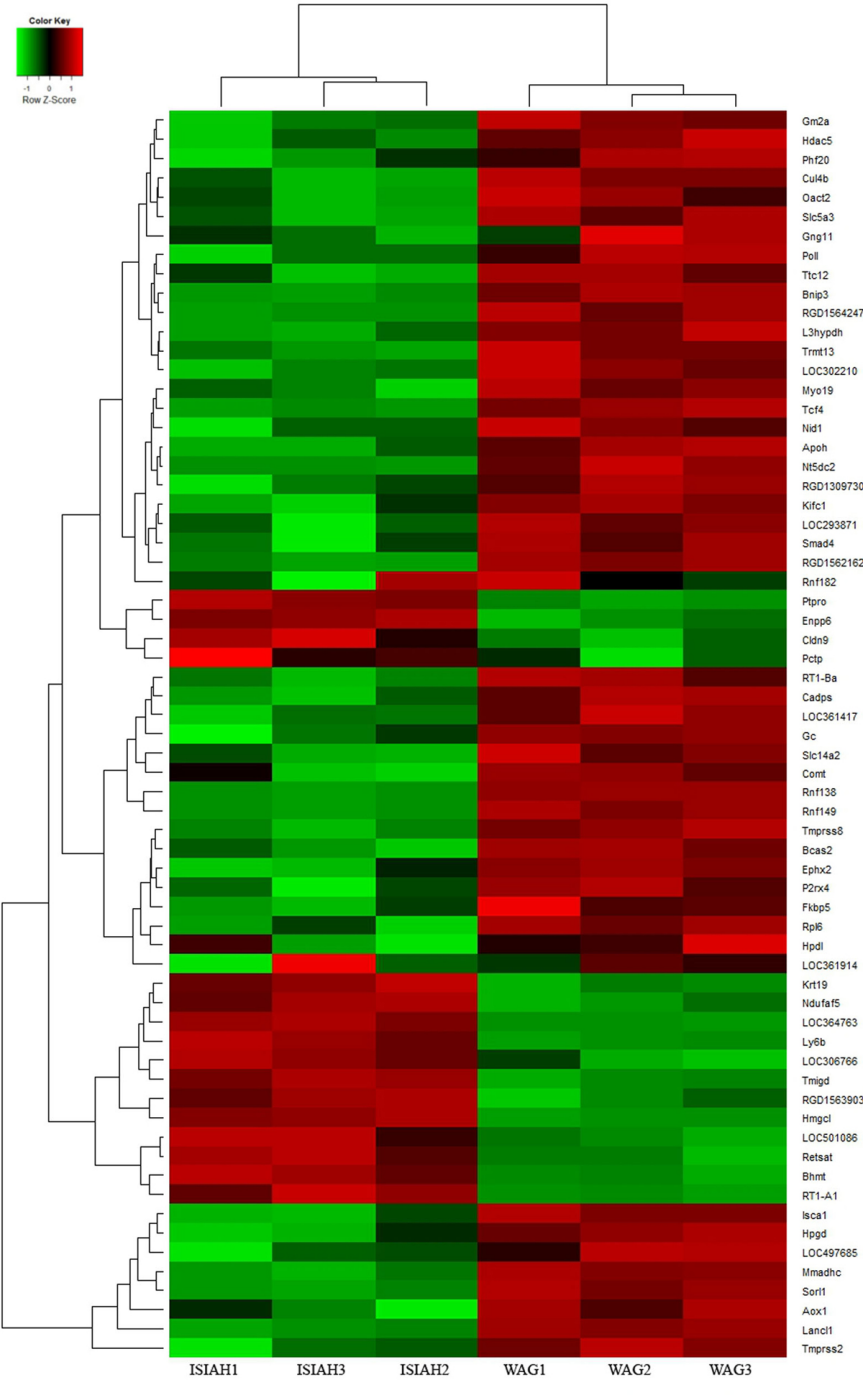
**Hierarchical clustering of the genes differentially expressed in renal medulla of hypertensive ISIAH and normotensive WAG rats**. Normalised gene expression is indicated by the row Z-score where red represents upregulated genes and green represents downregulated genes.

**Figure 3:**
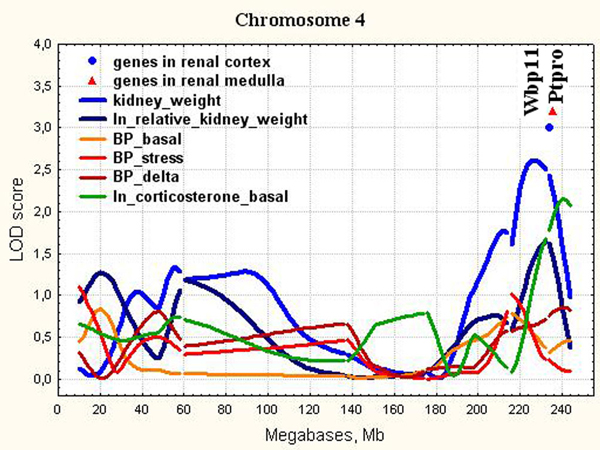
**The position of the differentially expressed genes in QTL for kidney weight on chromosome 4**. LOD score for kidney weight is 2.61. It exceeds 1% chromosome-wise threshold value 2.44.

**Figure 4:**
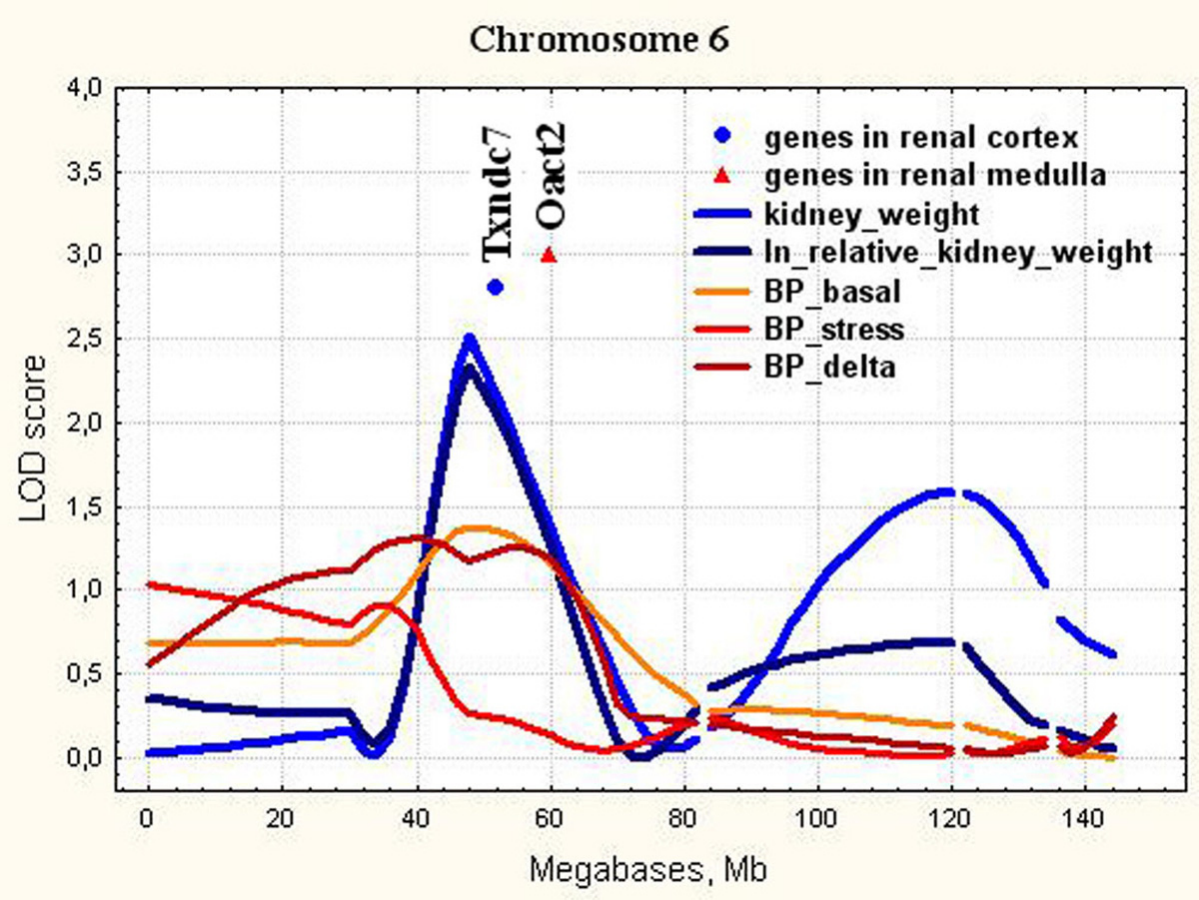
**The position of the differentially expressed genes in QTL for kidney weight and for relative kidney weight on chromosome 6**. LOD score for kidney weight is 2.52. It exceeds 1% chromosome-wise threshold value 2.40. LOD score for relative kidney weight is 2.34. It exceeds 1% chromosome-wise threshold value 2.14.

**Figure 5:**
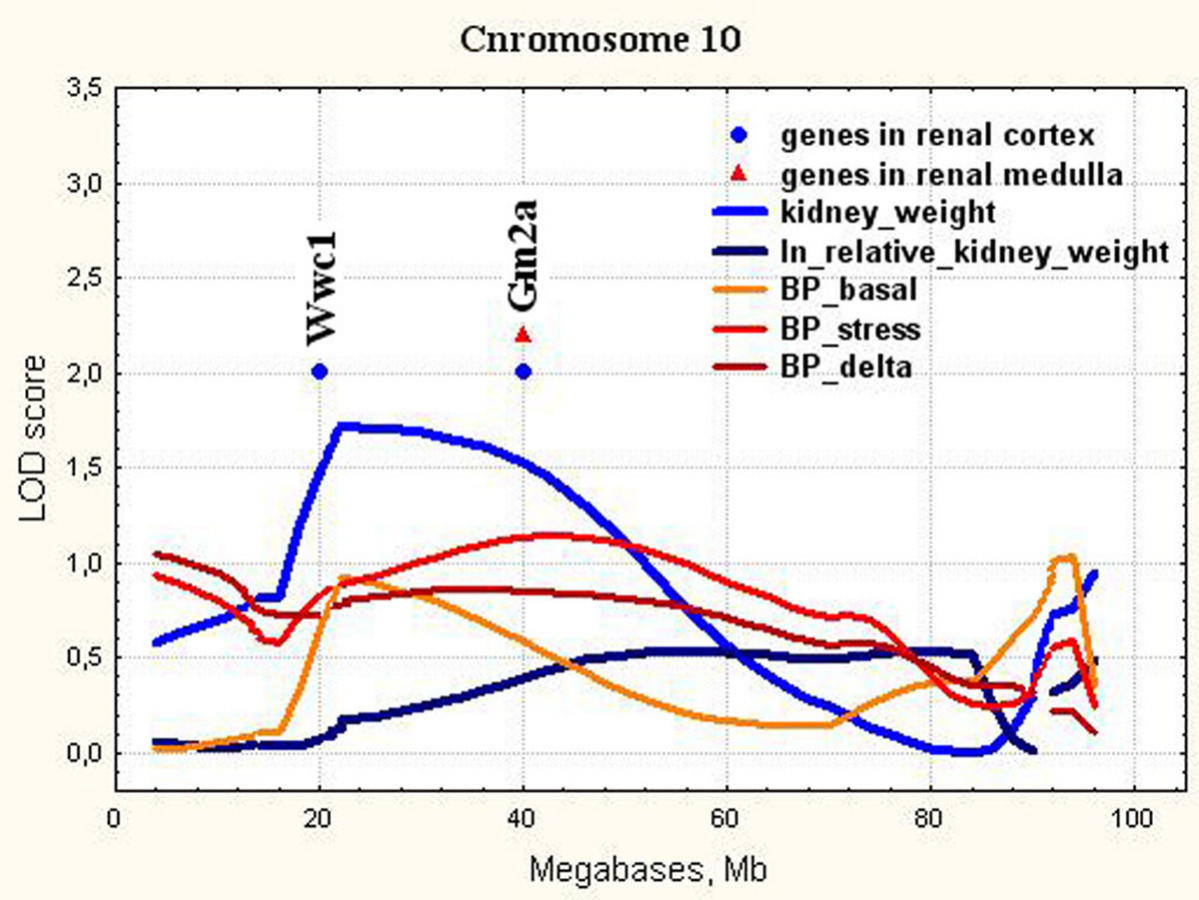
**The position of the differentially expressed genes in QTL for kidney weight on chromosome 10**. LOD score for kidney weight is 1.72. It exceeds 5% chromosome-wise threshold value 1.54.

**Figure 6:**
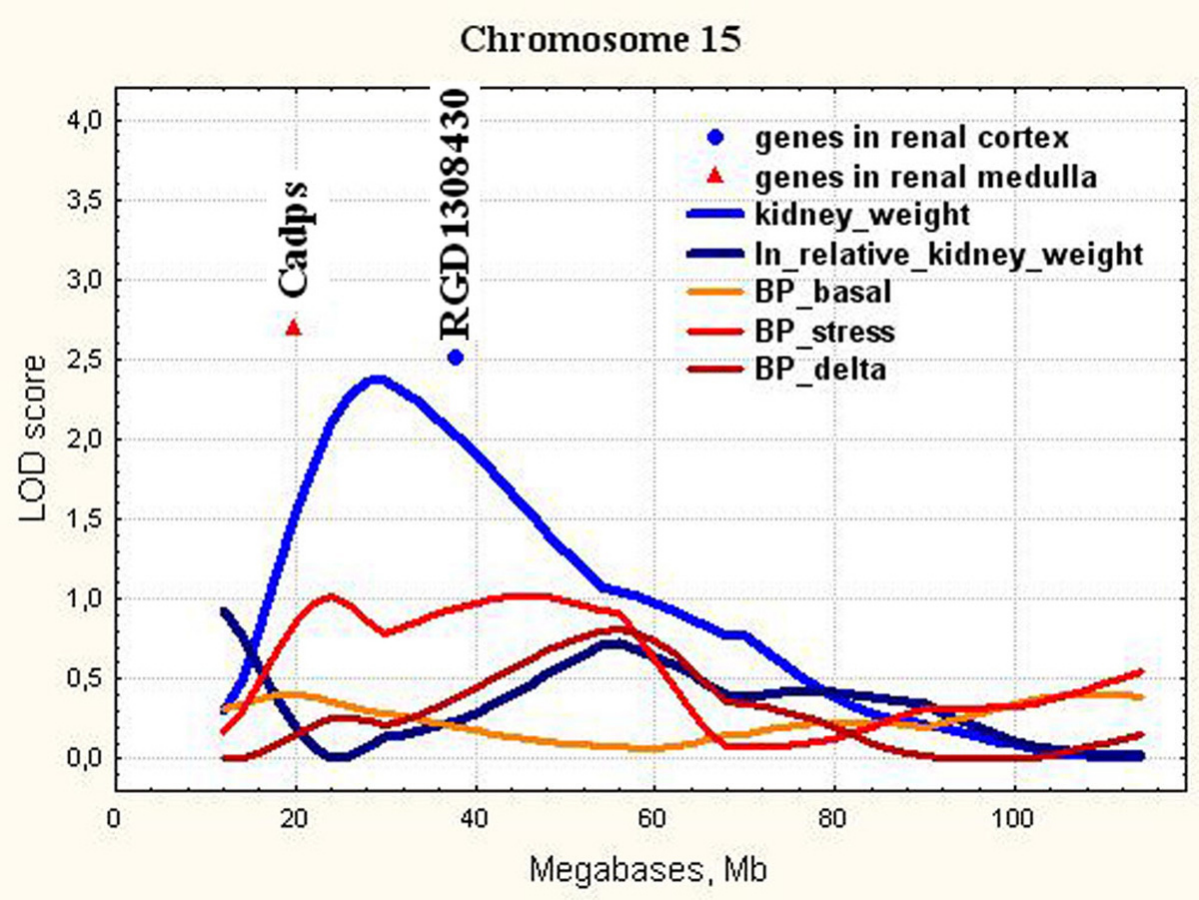
**The position of the differentially expressed genes in QTL for kidney weight on chromosome 15**. LOD score for kidney weight is 2.37. It exceeds 1% chromosome-wise threshold value 2.12.

**Figure 7:**
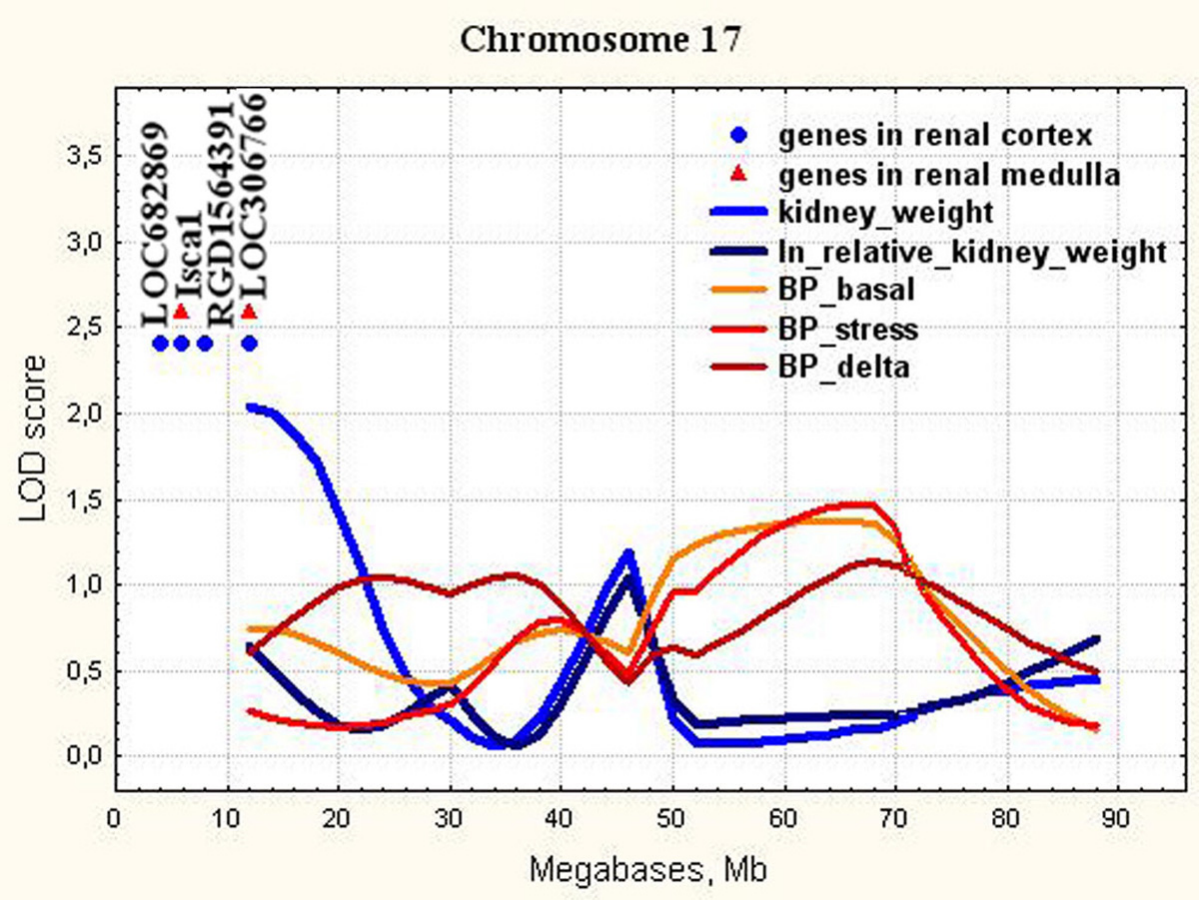
**The position of the differentially expressed genes in QTL for kidney weight on chromosome 17**. LOD score for kidney weight is 2.04. It is equal to 2.5% chromosome-wise threshold value.

**Figure 8:**
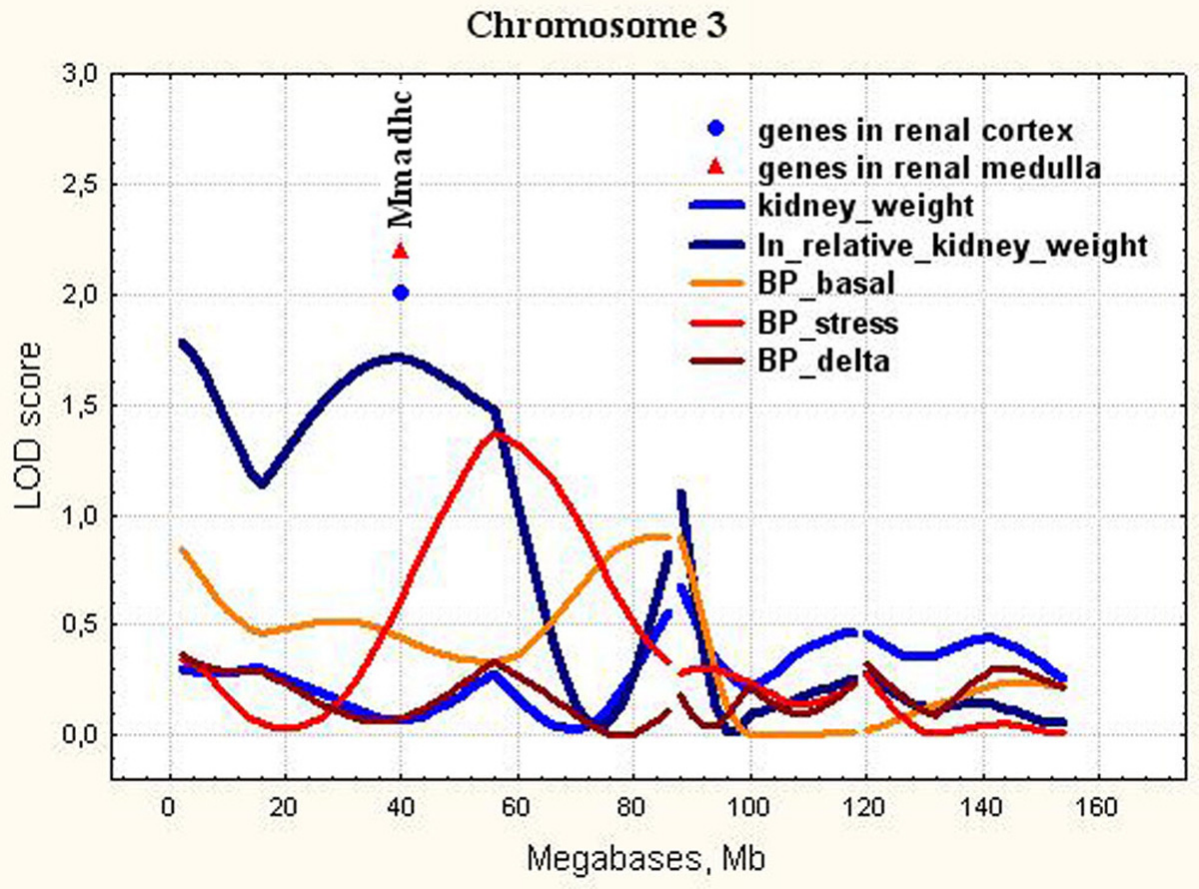
**The position of the differentially expressed genes in QTL for relative kidney weight on chromosome 3**. LOD score for relative kidney weight is 1.71. It exceeds 5% chromosome-wise threshold value 1.50.

**Figure 9:**
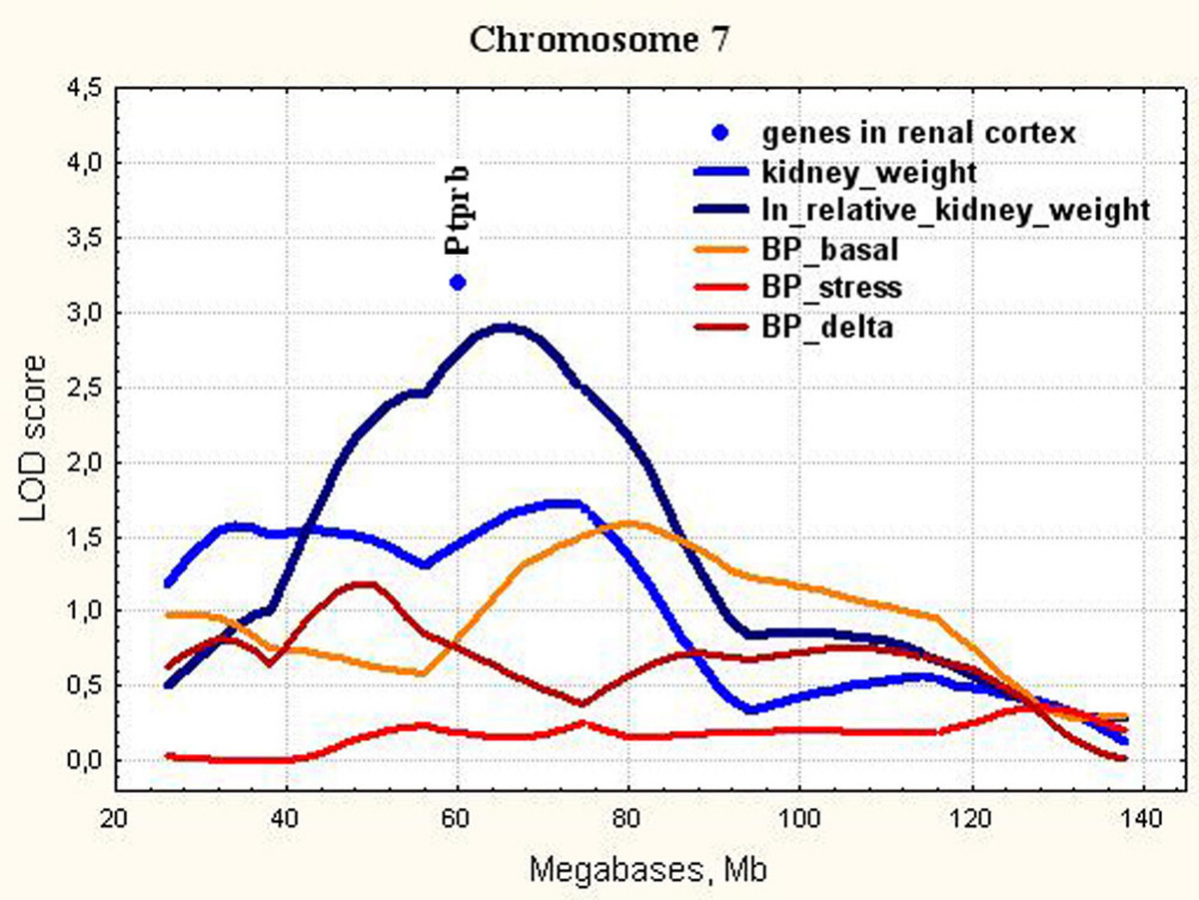
**The position of the differentially expressed genes in QTL for relative kidney weight on chromosome 7**. LOD score for relative kidney weight is 2.91. It exceeds 5% experiment-wise threshold value = 2.74.

Comparative analysis of gene expression profiling in kidney of hypertensive ISIAH and normotensive WAG rats revealed 126 differentially expressed genes in renal cortex and 65 differentially expressed genes in renal medulla [12]. The hierarchical clustering and heatmaps illustrating each individual's expression pattern in genes differentially expressed (p < 0,01) in kidney of hypertensive ISIAH and normotensive WAG rats are shown in Figures 1 and 2. In the present work we determined several differentially expressed genes (Table 1 Figures 3, 4, 5, 6, 7, 8, 9) mapped to genetic loci associated with the absolute and relative kidney weight described earlier for 6-month old F_2_(ISIAH × WAG) hybrid male rats [8]. It is considered that the determination of differentially expressed genes between selected lines of animals, and their localization within QTLs for the selected phenotype, dramatically increases the probability of identifying genes that contribute to that phenotype through differential expression [10,11,23]. It is understandable that both real target genes and genes located in loci just by chance could be found among these genes. The further discussion will help to discriminate between the differentially expressed genes located in QTL and to suggest the candidate genes in the loci for absolute and relative kidney weight which may be related to the structural and mass changes in hypertensive ISIAH kidney.

**Table 1:** Genes differentially expressed in ISIAH and WAG kidney and localized in QTL for absolute and relative kidney weight in 6-month old F_2 _(ISIAH × WAG) males

QTL			**Genes differentially expressed in ISIAH and WAG kidney**^∆^				
**Chr**.	**Peak marker (Mb)**	Confidence interval, * Mb	**Ratio** **ISIAH/ WAG**	**Acc.#**	**Symbol**	**Mb**	**Definition**

**kidney_weight**							

4	D4Rat68 (233.3)	204-242	0.37 0.56 (p<0.05) **0.36**	NM_001009661.1 NM_017336.1 **NM_017336.1**	Wbp11 Ptpro **Ptpro**	234.4 235.5 **235.5**	WW domain binding protein 11 Protein tyrosine phosphatase, receptor type, O **Protein tyrosine phosphatase, receptor type, O**

6	D6Rat143 (48.1)	42-62	0.56 **0.38**	XM_576132.1 **XM_001074910.1**	Txndc7 **Oact2**	52.3 **60.9**	Thioredoxin domain containing 7 **O-acyltransferase (membrane bound) domain containing 2**

10	D10Rat43 (22.3)	10-58	1.69 0.42 **0.38**	XM_220308.4 NM_172335.2 **NM_172335.2**	Wwc1 Gm2a **Gm2a**	20.5 40.3 **40.3**	WW and C2 domain containing 1 GM2 ganglioside activator **GM2 ganglioside activator**

15	D15Rat80 (30.3)	18-50	**0.28** 1.66	**NM_013219.1** XM_001054512.1	**Cadps** RGD1308430	**19.3** 37.2	**Ca++-dependent secretion activator** Similar to 1700123O20Rik protein

17	D17Rat107 (11.8)	0-24	1.84 0.51 **0.35** 0.08 6.54 **5.06**	XM_001061265.1 NM_181626.3 **NM_181626.3** XM_346945.2 NM_001014007.1 **NM_001014007.1**	LOC682869 Isca1 **Isca1** RGD1564391 LOC306766 **LOC306766**	5.1 7.5 **7.5** 7.8 12.7 **12.7**	similar to Golgi phosphoprotein 2 (Golgi membrane protein GP73), transcript variant 2 Iron-sulfur cluster assembly 1 homolog (S. cerevisiae) **Iron-sulfur cluster assembly 1 homolog (S. cerevisiae)** RGD1564391 (predicted) Hypothetical LOC306766 **Hypothetical LOC306766**

**ln_relative_kidney_ weight**							

3	D3Rat56-D3Rat130 (2.6-55.2)	0-62	0.37 **0.11**	NM_001004280.1 **NM_001004280.1**	Mmadhc **Mmadhc**	40.9 **40.9**	methylmalonic aciduria (cobalamin deficiency) cblD type, with homocystinuria **methylmalonic aciduria (cobalamin deficiency) cblD type, with homocystinuria**

6	D6Rat143 (48.1)	42-62	0.56 **0.38**	XM_576132.1 **XM_001074910.1**	Txndc7 **Oact2**	52.3 **60.9**	Thioredoxin domain containing 7 **O-acyltransferase (membrane bound) domain containing 2**

7	D7Rat51-D7Rat165 (54.6-73.5)	44-84	0.57	XM_235156.4	Ptprb	59.4	protein tyrosine phosphatase, receptor type, B (predicted)

*- the QTL boundaries were determined in the respective one LOD confidence interval. Mb - megabases.

∆-genes differentially expressed in renal cortex of ISIAH and WAG rats are given in regular type and genes differentially expressed in renal medulla of ISIAH and WAG rats are given in bold type letters.

### Genes in QTL for kidney weight

The QTL for kidney weight in the distal part of Chr.4 in ISIAH rats partially overlaps Kidney mass QTL 34 (Kidm34) (210-233 Mb) found in rats with Metabolic Syndrome X and increased relative kidney weight [24] and with the rat QTL Coreg2 for compensatory renal growth (CRG) (210-224 Mb) of the remnant kidney after unilateral nephrectomy [25]. However, the *Cacna1c *(216,6 Mb) gene suggested as a positional candidate for CRG in Coreg2 was not significantly expressed (Detection P-value < 0.05) in both kidney cortex and medulla of ISIAH and WAG rats. Two other differentially expressed genes, *Wbp11 *and *Ptpro*, have been located in QTL for kidney weight in ISIAH rats in the distal part of Chr.4 (Figure 3).

*Wbp11 *regulates mRNA processing and is involved in RNA splicing [26,27]. Its transcriptional activation is associated with enhanced expression of genes that regulate RNA processing, splicing, and degradation [28]. WBP11 was one of urinary polypeptides significantly down-regulated and specific for essential hypertension with left ventricular diastolic dysfunction that subsequently distinguished hypertensive patients with overt heart failure from healthy controls [29]. The QTL for kidney weight in the distal part of Chr.4 does not overlap with loci for blood pressure traits in ISIAH rats but overlaps with the locus where the ISIAH alleles significantly increase the basal level of corticosterone (Figure 3) [13]. Corticosterone may induce the formation of reactive oxygen species [30] and development of adaptive response to oxidative stress may influence the mRNA processing [31] causing both the induction of stress-response genes and inhibition of gene transcription [32]. So, the down-regulation of Wbp11 in ISIAH kidney may be relevant to changes in transcriptional level of many genes found in current study but probably doesn't have direct effect on the kidney weight or structural changes in kidney histology related to the trait.

*Ptpro *(or GLEPP1, glomerular epithelial protein 1) is a receptor tyrosine phosphatase expressed on the apical cell surface of the glomerular podocyte [33]. The GLEPP1 (Ptpro) receptor plays a role in regulating the glomerular pressure/filtration rate relationship through an effect on podocyte structure and function. Podocytes are specialized epithelial cells with delicate interdigitating foot processes which cover the exterior basement membrane surface of the glomerular capillary. It was demonstrated that glomerular enlargement is associated with podocyte hypertrophy, podocyte stress, and the decrease in *Ptpro *expression in the aging Fischer 344 rats known to develop spontaneous glomerulosclerosis with age [34]. *Ptpro *is localized in QTL for renal function (Rf13) (224-248 Mb) found in hypertensive salt-sensitive rats given a high-salt diet (8% NaCl) and associated with change in renal blood flow rate [35]. Ptpro-/- mice had an amoeboid rather than the typical octopoid structure seen in the wild-type mouse podocyte and blunting and widening of the minor (foot) processes. *Ptpro^-/- ^*mice had reduced glomerular filtration function and a tendency to hypertension [36]. The extensive loss of GLEPP-1 was found in patients with focal segmental glomerulosclerosis and collapsing glomerulopathy [37]. GLEPP1 expression is considered to be a useful marker of podocyte injury [38]. *Ptpro *downregulation in ISIAH kidney may be responsible for the podocyte histological changes. It may be considered as a candidate gene for the kidney histological changes leading to the increased kidney weight in ISIAH rats.

Another locus mapped on chromosome 6 was the same for both kidney weight and relative kidney weight traits in ISIAH rats (Figure 4). This locus overlaps with the rat QTL Coreg1 for compensatory renal growth (CRG) (51-70 Mb) of the remnant kidney after unilateral nephrectomy [39]. In our study, the QTL on chromosome 6 contained 2 genes differentially expressed in hypertensive and normotensive kidney. These were *Txndc7 *in renal cortex and *Oact2 *in renal medulla.

*Txndc7 *(or *Pdia6*), is one of the endoplasmic reticulum (ER) resident genes (proteins) that control ER functions and are responsive to cellular stress, including metabolic and oxidative stress. ER stress can be triggered by hypoxia, nutrient deprivation, perturbation of redox status, aberrant Ca2+ regulation, viral infection, failure of posttranslational modifications, and increased protein synthesis and/or accumulation of unfolded or misfolded proteins in the ER [40]. Gain- and loss-of-function studies showed that PDIA6 protected cardiac myocytes against simulated ischemia/reperfusion-induced death and this protection is dependent on the oxidoreductase activity of PDIA6 [41].

ER stress is a pathologic mechanism in a variety of chronic diseases. ER stress inhibition reduces cardiac damage and improves vascular function in hypertension [42]. The position of *Txndc7 *corresponds to genome region where small QTL for basal blood pressure may be suggested (Figure 4). According to established statistical approaches this locus for blood pressure can't be considered as significant or even suggestive. The locus is characterized by LOD score 1.37, and is accounting 4,9% of the trait variability. From the other side, some researchers agree that many small QTL are smeared across the genome and many small QTL effects control polygenic trait variation [43-45]. Based on this, we may suggest that the decreased expression of the *Txndc7 *in ISIAH kidney probably doesn't affect the kidney weight but may cause the enhanced cellular ER stress, which may contribute to vascular complications and development of stress-induced hypertension in ISIAH rats.

*Oact2 *(or *Mboat2*), O-acyltransferase (membrane bound) domain containing 2 is acyltransferase, which mediates the conversion of lysophosphatidylcholine into phosphatidylcholine. Phosphatidylcholine is a major component of cellular membranes and is the most abundant phospholipid in kidney cortical tubules [46]. Its increased biosynthesis was found during renal growth following unilateral nephrectomy [47]. *Oact2 *was identified as one of the genes with high predictive power (87%) in segregating malignant from benign lesions [48]. *Oact2 *is localized in chromosomal region where the QTL for kidney dilation (Kiddil4) (31.9-94.3 Mb) associated with the degree of dilation of the renal pelvis in rats with congenital hydronephrosis [49] and QTL for renal function (Rf14) (44-90 Mb) associated with the salt-loaded renal blood flow [35] were found. The presence of two ISIAH alleles in the QTL for absolute and relative kidney weight on Chr.6 in F_2 _(ISIAH × WAG) hybrid males caused the significant decrease in kidney weight and in relative kidney weight [8](Supplement, Table 4). Based on that we may suggest that *Oact2 *may be considered as a candidate gene in QTL and its downregulation in ISIAH renal medulla may play protective role against the hyperplastic process in hypertensive kidney.

*Wwc1 *(Chr.10, Figure 5) encodes KIBRA protein, which is predominantly expressed in the kidney and brain in the adult organism [50]. In the kidney, KIBRA is expressed in glomerular podocytes, in some tubules, and in the collecting duct [51]. KIBRA regulates epithelial cell polarity by suppressing apical exocytosis through direct inhibition of aPKC kinase activity [52]. In renal podocytes, KIBRA/WWC1 has an impact on targeted cell migration and links polarity complexes to the cytoskeleton [51]. KIBRA regulates precise mitosis [53], cell-cycle progression [54], and it is known as an upstream regulator of tumor suppressor Hippo pathway that regulates cell proliferation and apoptosis [55]. Hippo signaling is an evolutionarily conserved signaling pathway that controls organ size from flies to humans [56]. Hippo-Yap pathway has been shown to play a key role in controlling organ size, primarily by inhibiting cell proliferation and promoting apoptosis. Overexpression and knockdown studies demonstrate that KIBRA promotes the collagen-stimulated activation of the MAPK cascade that is involved in various cellular functions, including cell proliferation, differentiation and migration [57]. KIBRA knockdown impairs cell migration and proliferation in breast cancer cells [58]. *Wwc1 *is localized in QTL for relative kidney mass (Kidm21) found earlier in the Lyon hypertensive rats [59]. The presence of two ISIAH alleles in the QTL for absolute and relative kidney weight on Chr.10 in F_2 _(ISIAH × WAG) hybrid males caused the significant increase in kidney weight [8]. Based on this, we may suggest that *Wwc1 *may be considered as a candidate gene in QTL on Chr.10 for kidney weight and its upregulation in ISIAH renal cortex may play important role in the renal mass gain.

*Gm2a *(Chr.10, Figure 5) is a lysosomal protein related to lipid transporter activity [60]. It may participate in vesicular transport in collecting duct intercalated cells [61] but nothing is known about its influence on the renal mass.

*Cadps *(Chr.15, Figure 6) is a Ca++-dependent secretion activator. It is required for optimal vesicle exocytosis in neurons and endocrine cells [62]. It regulates catecholamine release from neuroendocrine cells through the interaction with dopamine D2 receptor [63]. The deletion of CADPS alleles causes the deficit in catecholamine secretion [64]. Dopamine receptors of DA-2 subtypes are localized in sympathetic nerve terminals innervating the renal blood vessels. Some selective DA-2 receptor agonists are effective antihypertensive agents [65]. CADPS is one of the positional candidate genes in human blood pressure quantitative trait loci [66]. Genetic down-regulation of genes related to the adrenergic system (including *Cadps*) might play a role in splanchnic vasodilation of portal hypertension [67]. *Cadps *location corresponds to chromosomal region characterized by a very low LOD score 1.01 for blood pressure level at stress in ISIAH rats (Figure 6). But, as we agree that many small QTL effects control polygenic trait variation, we suggest that *Cadps *downregulation may be a part of adaptive mechanism against BP elevation at stress in ISIAH rats.

*Isca1 *(Chr.17, Figure 7) is implicated in the biogenesis of iron-sulfur clusters. Iron-sulfur clusters are integral parts of proteins that participate in oxidation-reduction reactions and catalysis [68,69]. It is known, that iron is essential for healthy life and is involved in numerous metabolic processes including cell growth and proliferation [70]. However, no relations between *Isca1 *and kidney weight were reported.

Several other genes with differential expression and unknown functions were also detected in the QTL for kidney weight on Chr.15 and Chr.17 (Figures 6 - 7). The further studies are needed to define their functions which probably may be related to increased kidney weight and structure abnormalities in ISIAH rats.

### Genes in QTL for relative kidney weight

In the current study, the QTL for relative kidney weight on Chr.3 contained the only differentially expressed gene (Figure 8). It was *Mmadhc *gene. Its expression was significantly decreased in hypertensive ISIAH kidney.

*Mmadhc *is related to cobalamin (Cbl, vitamin B12) transport and metabolism, the defects of which may cause methylmalonic aciduria, homocystinuria, or both [71,72]. Patients with methylmalonic aciduria often develop chronic renal failure (CRF) [73]. Kidney weight per unit of body weight was significantly greater in the Cbl-deficient rats compared with the two Cbl-sufficient control groups [74]. *Mmadhc *is localized in chromosomal region where the QTL for kidney mass (Kidm13) was found in the Lyon hypertensive rats [59]. The presence of two ISIAH alleles in the QTL for relative kidney weight on Chr.3 in F_2 _(ISIAH × WAG) hybrid males caused the significant increase in relative kidney weight [8](Supplement, Table 4). We suggest that decrease in *Mmadhc *expression may contribute to the increase in relative kidney weight in ISIAH rats due to a possible abnormal cobalamin transport and metabolism.

The chromosome 6 was characterized by QTL common for absolute and relative kidney weight (Figure 4). The genes differentially expressed in ISIAH and WAG kidney and located in QTL on Chr.6 were discussed above.

*Ptprb *(Chr.7, Figure 9) is a receptor protein tyrosine phosphatase beta. It is a receptor for heparin affin regulatory peptide (HARP), which is a growth factor that has a potent role in tumor growth and angiogenesis. RPTPβ down-regulation interrupts HARP signaling in human umbilical vein endothelial cells and abolishes its biological activity on cell migration and differentiation [75]. *Ptprb *expression mediates deafferentation-induced synaptogenesis [76] and regulates sodium channel modulation in brain neurons [77].

The earlier studies have demonstrated adrenergic nerve terminals in direct contact with basal membranes of mammalian renal tubular epithelial cells. The stimulation of renal sympathetic nerves produces an increase in renal tubular sodium reabsorption without alterations in glomerular filtration rate, renal blood flow, or intrarenal distribution of blood flow [78]. As soon as the statistically significant plasma sodium increase was found in ISIAH rats as compared to normotensive WAG [79], we may suggest that the decreased expression of *Ptprb *in ISIAH kidney may be adaptive against the excessive renal sodium retention but probably doesn't influence the kidney weight.

In the current study, we showed that all loci found for absolute and relative kidney weight didn't overlap with significant or suggestive loci for BP traits (Figure 3, 4, 5, 6, 7, 8, 9). So, the genes differentially expressed in ISIAH and WAG kidneys and located in these QTL regions associated with absolute and relative kidney weight shouldn't substantially influence the BP level in the 6 month-old ISIAH rats. However, we consider that in some cases small effects may be suggested and that is in a good agreement with the recent insights into genetic architecture of complex diseases [80]. These loci, one by one, have a little association with the blood pressure. However, one can expect that the summation of their effects in a whole genome can result in much more higher levels of the association.

Earlier we described several loci common for relative kidney weight and blood pressure traits in QTL analysis of F_2 _(ISIAH × WAG) hybrid males aged 3-4 month old [8]. We suggested the important role of kidney function in early stage of hypertension manifestation in ISIAH rats and switching to other mechanisms leading to genetic control of BP level in the 6-month-old rats. It was shown that the significant QTL on chromosome 1 was common for arterial blood pressure at rest and under the emotional stress conditions and for relative spleen weight in the 6-month-old F_2_(ISIAHxWAG) rats. These results suggest that the manifestation of the stress-sensitive arterial hypertension in ISIAH rats of that age may be under the genetic control of the determinants related to the spleen function [81]. This dynamic change of QTL effects during a time course might reflect the process of stress-sensitive hypertension development.

Earlier some authors reported that a phenotype having some genetic component may be affected by different genetic loci at different age. It was considered highly plausible and was shown in different organisms: rats [82,83], chicken [84], humans [85]. The dynamic change of QTL effects during the time course of growth points out that early and late growth, at least to some extent, have different genetic regulation [84].

The distinct kidney mass QTLs independent of those controlling BP were found earlier in studies of different models of hypertensive rats [2,86]. These and our studies suggest that kidney mass can be controlled by physiologic mechanisms different from those responsible for BP. As soon as the kidney mass has been viewed as a significant risk factor for the progression of renal diseases [87] the discovery of individual kidney mass QTLs may help to identify the mechanisms underlying renal hypertrophy independent of hypertension.

## Conclusion

The differentially expressed genes found in QTL may relate not only to the traits under study, but to other interstrain differences as well. However, the QTL/microarray approach and the knowledge-driven filtering of the list of candidates helped to determine several positional candidate genes in the QTL for absolute and relative kidney weight, which may be related to the structural and mass changes in hypertensive ISIAH kidney. These were *Mmadhc, Ptpro, Oact2 *and *Wwc1*.

The rationale behind QTL/microarray studies is that causative genes may have polymorphisms causing differences in their level of expression that translate into varying amounts of mRNA and ultimately varying amounts of functional proteins, leading to observable phenotypes [88]. From the other side the differential transcription of the QTL-associated candidate genes may be a result of the trans-regulation mechanism.

The further experimental validation of causative genes and detection of polymorphisms will provide opportunities to significantly advance our understanding of the underlying nature of structural and mass changes in hypertensive ISIAH kidney.

## Competing interests

The authors report no conflicts of interest. The authors alone are responsible for the content and writing of the paper.

## Authors' contributions

OR carried out the QTL analysis, performed the statistical analysis and drafted the manuscript. SS carried out the QTL analysis, participated in the statistical analysis and drafted the manuscript. LK performed hierarchical cluster analyses and heatmaps construction. AM conceived of the study, participated in its design and coordination and helped to draft the manuscript.

## Declarations

Publication of this article has been funded by the RSF 14-14-00269 grant.

This article has been published as part of *BMC Genetics *Volume 16 Supplement 1, 2015: Selected articles from the IX International Conference on the Bioinformatics of Genome Regulation and Structure\Systems Biology (BGRS\SB-2014): Genetics. The full contents of the supplement are available online at http://www.biomedcentral.com/bmcgenet/supplements/16/S1.
